# Comprehensive report of an *Enterococcus cecorum* infection in a broiler flock in Northern Germany

**DOI:** 10.1186/s12917-014-0311-7

**Published:** 2014-12-24

**Authors:** Arne Jung, Silke Rautenschlein

**Affiliations:** Clinic for Poultry, University of Veterinary Medicine Hannover, Buenteweg 17, D-30559 Hannover, Germany; Buenteweg 17, D-30559 Hannover, Germany

**Keywords:** *Gallus gallus domesticus*, Chicken, Broiler, Infection, Septicemia, Osteomyelitis, Pericarditis, Hepatitis, *Enterococcus cecorum*, Pathogenesis, Route of infection, Condemnation rate

## Abstract

**Background:**

*Enterococcus cecorum* is considered as an emerging pathogen in poultry and can cause substantial losses in broiler flocks. Femoral head necrosis and spondylitis were described as the main pathological changes in infected chickens. Nevertheless, little is known about the pathogenesis of *Enterococcus cecorum* infection in broilers. This report shows for the first time the whole course of disease over an entire growing period including repeated necropsies and subsequent microbiological investigations.

**Case presentation:**

In a flock of 18200 broilers, a decrease in flock uniformity was detected from 14 days post hatch onwards with affected chickens showing lameness and an increase in flock mortality up to 7.22% at day 33 post hatch. In the first 3 weeks post hatch, pericarditis and hepatitis were found as the main pathological changes in 27.6% and 9.8% of the examined broilers respectively. Femoral head necrosis and vertebral osteomyelitis were detected in the last week of the growing period with 10.3% and 2.3% respectively. Heart, liver, spleen, yolk sac and vertebral column of 59 broilers with pathological changes were subjected to bacteriological analysis. *Enterococcus cecorum* was isolated from 23 birds (39%), the first broiler was already positive at day 3 post hatch in the yolk sac. Additionally, 9.75% of the broilers were rejected at the slaughterhouse primarily because of pathological changes. The investigated broiler cycle had by far the best footpad score compared to 7 cycles before and 4 cycles after the *Enterococcus cecorum* infection at the same farm.

**Conclusions:**

Bacteraemia and generalized infection appear to be important steps in the pathogenesis of *Enterococcus cecorum* infection in broilers. Furthermore, this disease causes economic losses for the farmer not only due to an increase in flock mortality, but probably also through substantially higher condemnation rates at the slaughterhouse. It was speculated that the broilers were infected via the respiratory tract as this flock had lower footpad scores likely the result of drier litter. The latter may have led to higher dust concentrations and thus airborne *Enterococcus cecorum*.

## Background

*Streptococcus cecorum* was first isolated in 1983 from the caeca of healthy chickens [1]. In 1989 it was reclassified as *Enterococcus cecorum* (EC) in the genus *Enterococcus* [2]. In this genus, currently 54 species are described (http://www.bacterio.net) and most of them are part of the intestinal flora of mammals and birds or can be isolated from environmental sources [3]. EC does not show certain characteristics traditionally considered to be typical for the genus *Enterococcus* including growth at 10°C, at 6.5% NaCl concentration and on certain *Enterococcus* selective media [4]. So far, EC has been detected in the intestinal tract of healthy horses, cattle, pigs, dogs, cats, chickens, canaries, pigeons, turkeys and Muscovy ducks [5-9]. In 2002, EC-associated disease outbreaks in broiler flocks were reported for the first time [10,11]. Further reports from broiler and broiler breeder flocks indicate the growing importance of EC infections for the poultry industry [12-17]. In Germany, EC is considered to be one of the most important bacterial pathogens in broilers (personal communication with other poultry veterinarians). EC associated disease outbreaks were also reported from ducks in Germany [18,19] and EC infection was successfully reproduced experimentally in Pekin ducklings [20], demonstrating the ability of EC to induce disease in other birds than chickens.

Typical clinical signs of EC infection in infected broilers and broiler breeders are seen between 5 to 10 weeks of age with a marked increase in flock mortality [15,21]. Affected broilers and broiler breeders exhibit osteomyelitic changes of the femoral heads and the vertebral column causing lameness and hind-limb paresis.

So far, little is known about the pathogenesis of EC in broilers. The available reports mostly lack detailed data because they usually presented results from animals selected at one time point during the course of disease. This case report demonstrates a detailed follow up investigation of an EC infection in a German broiler flock from placement of the broilers until slaughter, including data from the slaughterhouse.

## Case presentation

### Animals, environment, treatment and sampling

The affected flock consisted of 18200 broilers (Ross 308), equally divided into two groups of 9100 birds, which were housed in the two compartments (C1 and C2) of a broiler house, separated by a lightweight wall but connected by a door for personnel. One-day-old broilers, vaccinated in the hatchery with Poulvac® IB Primer (Zoetis), administered as coarse spray, were obtained from BWE hatchery (Visbek, Germany), placed on wood shavings and fed with standard 3-phase pelleted feed. The starter feed contained Narasin (60 mg/kg feed) and Nicarbazin (125 mg/kg feed), grower feed was used from day 11 post hatch (ph) and contained Monensin-Sodium (100 mg/kg feed), finisher feed was used from day 29 ph and was free from anticoccidials. Birds in the two compartments were treated equally, apart from an *E. coli*-vaccination. Broilers in C1 were vaccinated at the day of arrival with Poulvac® E. coli (Zoetis Deutschland GmbH, Berlin, Germany), administered as coarse spray, while broilers in C2 were not vaccinated. All broilers were vaccinated as follows: day 11 ph Poulvac® ND Hitchner B1 (Zoetis Deutschland GmbH, Berlin, Germany), day 13 ph AviPro® Precise (LAH, Cuxhaven, Germany) day 18 ph Poulvac® IB Primer (Zoetis). All broilers were treated with KoniCalPhos (Konivet GmbH, Essen, Germany) and Agivit AD_3_EC (Mepro, Vechta, Germany) via drinking water from day 6 to day 11 ph. From day 24 to day 29 ph, all broilers were medicated against EC infection with Octacillin 800 mg/g (Albrecht GmbH, Aulendorf, Germany) at a dosage of 20 mg amoxicillin per kg bodyweight via the drinking water. Maximum stocking rate at the last day before slaughter was 39 kg/m^2^ according to German regulations.

### Clinical history

The broiler flock was monitored from placement until day 33 ph, transported in the following night and slaughtered in the morning of day 34 ph. From 14 dph a decrease in flock uniformity was detected in both compartments with some broilers growing less than the rest of the flock. Additionally, some of these animals showed reluctance to move. From 21 dph onward, the majority of affected broilers showed lameness and the difference in weight with healthy birds became more obvious. These signs persisted until the end of the growing period. The mortality in the broiler flock was 1.03% at 7 dph, 2.25% at 14 dph, 2.97% at 21 dph, 3.75% at 28 dph and 7.22% at day 33 ph and the mean daily mortality was 0.22%. Overall mortality in C1 was 5.84% and 8.59% in C2. The presented data include dead animals and broilers which had to be euthanized for animal welfare reasons. The mean mortality for the last 4 cycles of the farm, which were cycles without disease problems, was 3.54%.

### Gross pathology

**Figure 1:**
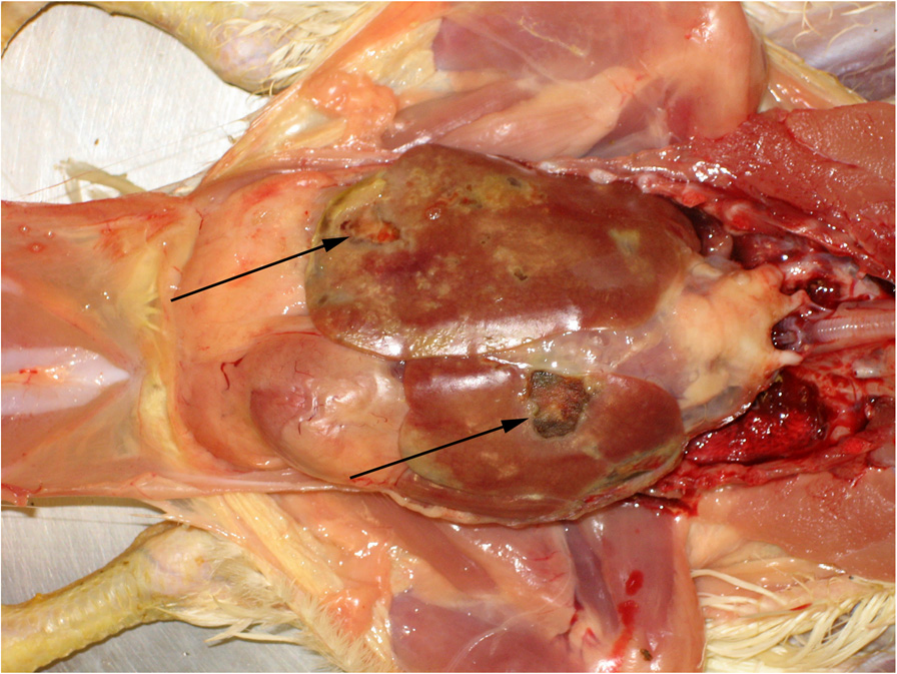
**Pericarditis and fibrinous hepatic necrosis (arrows) of an** ***Enterococcus cecorum*** **infected broiler, 24 days post hatch.**

**Figure 2:**
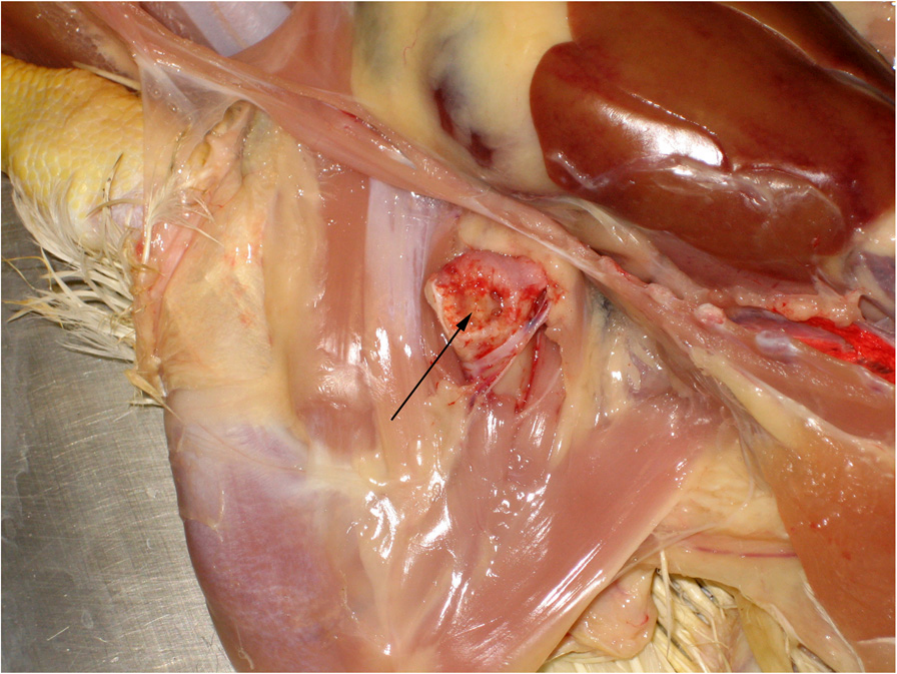
**Femoral head necrosis with mucopurulent exudate (arrow) of an** ***Enterococcus cecorum*** **infected broiler, 31 days post hatch.**

**Figure 3:**
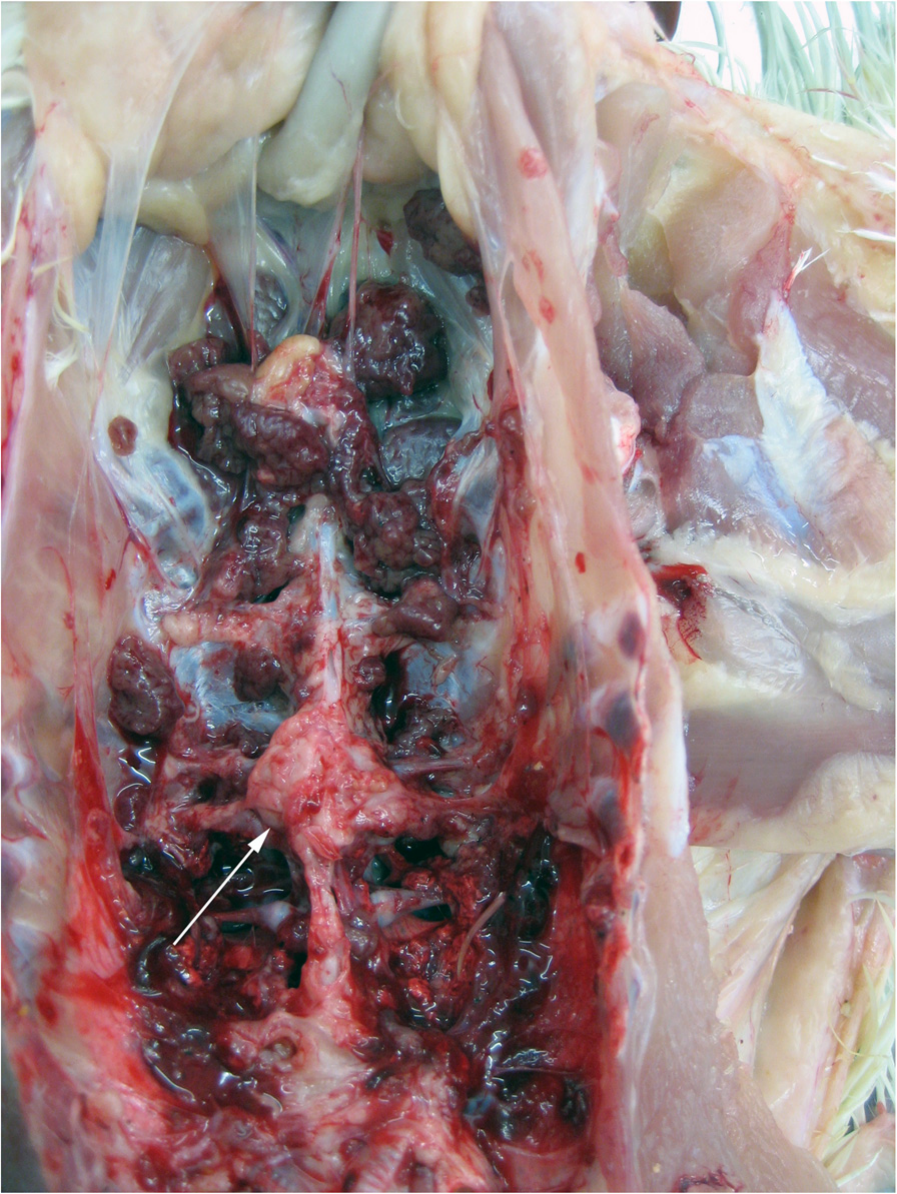
**Nodular enlargement of the caudal thoracic vertebrae (arrow) of an** ***Enterococcus cecorum*** **infected broiler, 31 days post hatch.**

**Figure 4:**
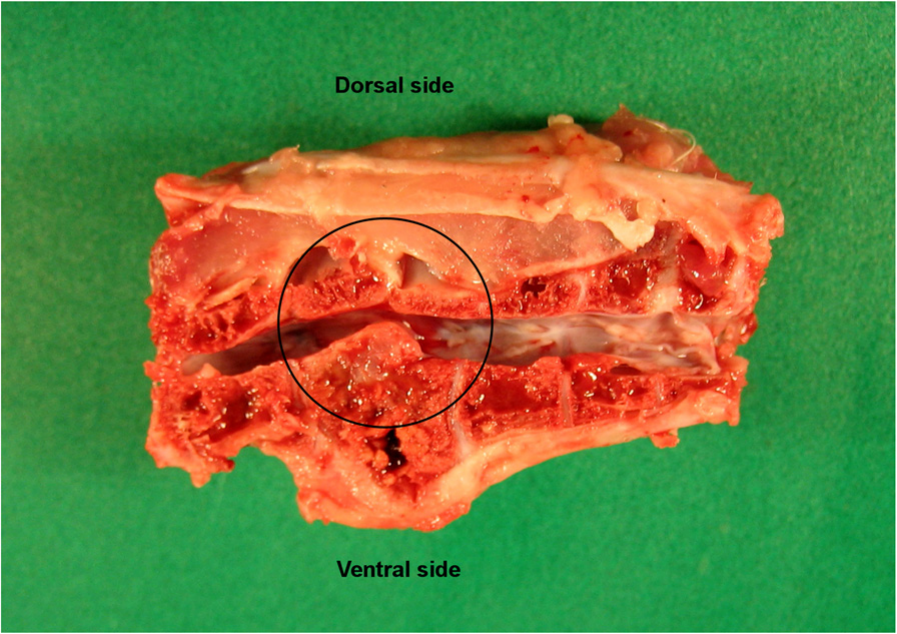
**Sagittal section of the vertebral column of an** ***Enterococcus cecorum*** **infected broiler, 31 days post hatch, spinal cord removed.** The osteomyelitic lesion leads to dorsal displacement of the vertebral canal and compression of the spinal cord (circle).

At days 3, 6, 10, 13, 17, 20, 24, 27 and 31 ph all dead and moribund broilers from the flock were submitted for necropsy, 174 animals in total (Table 1). At 3 dph, the majority of the examined chickens showed omphalitis. Beginning at day 6 ph, broilers developed pericarditis and hepatitis, most affected chickens showed enlarged livers of brown color in comparison to purple-red livers of healthy broilers. From 20 dph onward, some livers had also necrotic areas of several cm in diameter (Figure 1). Osteomyelitic changes of femoral heads (Figure 2) and the vertebral column (Figures 3 and 4) were not found before 17 and 27 dph respectively. The major pathologic lesion in examined broilers was pericarditis, which was found in 27.6% of the birds, followed by femoral head necrosis (10.3%), hepatitis and omphalitis (both 9.8%) and vertebral osteomyelitis (2.3%).

**Table 1:** **Macroscopic lesions and isolation of** ***E. cecorum*** **(EC) from examined** ^**A**^ **broilers**

**dph** ^**B**^	**Number of broilers**	**Macroscopic lesions**					
		**Omphalitis**	**Pericarditis**	**Hepatitis/hepatic necrosis**	**Femoral head necrosis**	**Vertebral osteomyelitis**	**EC positive animals** ^**C**^
3	18	15	0	0	0	0	1^D^/4
6	30	2	4	1	0	0	2/3
10	23	0	1	1	0	0	2/6
13	22	0	7	0	0	0	4/5
17	23	0	4	3	1	0	4/9
20	16	0	7	2	0	0	1/9
24	18	0	11	7	6	0	4/11
27	16	0	8	3	4	2	3/6
31	8	0	6	0	7	2	2/6

^A^At days 3, 6, 10, 13, 17, 20, 24, 27 and 31 post hatch all dead and moribund broilers from the flock were submitted for necropsy. ^B^Days post hatch. ^C^EC positive animals/total number of broilers subjected to bacteriological analysis. Samples of heart, liver and spleen from broilers with pathological changes were collected and processed for bacteriological examination. At 3 dph, heart, liver and yolk sac were collected. At 27 and 31 dph, an additional swab sampling of the vertebral column was done. Broilers with EC isolation from at least one organ were considered EC positive. ^D^Isolated from yolk sac.

### Post mortem examinations at the slaughterhouse

After transport of the broilers to the slaughterhouse and slaughtering of the animals, official post-mortem examinations of all broilers were conducted. 9.75% of the slaughtered broilers were rejected, most of them due to cellulitis (5.96% of slaughtered broilers) and generalized disease (2.75%; Table 2). Considering the total number of rejected animals (9.75%) 3.33% was allocated to section C1 and 6.42% to section C2. In comparison, the mean number of rejected broilers in the last 5 cycles at the same farm was 2.23%. The mean weight of the processable broilers, without the rejected animals, was 1994 g. Of the last 5 cycles at the farm, 3 cycles also lasted 33 days. The mean weight of the processable broilers in these 3 cycles was 1975 g.

**Table 2:** **Causes of rejection of carcasses from the EC infected broiler flock at the slaughterhouse** ^**A**^

	**Number of rejected broilers**	**% of slaughtered broilers (% of slaughtered broilers in the last 5 cycles** ^**B**^ **)**
Cellulitis, breast blisters	990	5.96 (1.29)
Ascites, polyserositis	157	0.94 (0.34)
Generalized disease (organ findings)	457	2.75 (0.38)
Organoleptic anomalies (colour, odor, consistency)	2	0.01 (0.05)
Emaciated animals/runts	7	0.04 (0.02)
Carcass soiling, faecal or other contaminations	0	0.00 (0.00)
Hematoma, traumata, cicatrisation	0	0.00 (0.05)
Other pathological changes	0	0.00 (0.00)
Insufficient bleeding	0	0.00 (0.00)
Processing damages	8	0.05 (0.10)
**Total**	**1621**	**9.75 (2.23)**

^A^Official examinations according to EU regulation 854/2004.

^B^The mean percentage of rejected broilers in the last 5 cycles of the farm is listed for comparison.

Footpads of all broilers were assessed with an automated camera system. The assessment is based on color and size of the lesions. The area of the footpads was determined and divided into pixels. The proportion of dark skin (representing footpad lesions) within the area designated as the footpad was measured and used for categorizing the severity of footpad dermatitis. Footpads were scored according to the number of dark pixels from score 1 to score 4, with 0–5 dark pixels as score 1, 6–19 dark pixels score 2, 20–44 dark pixels score 3 and 45–100 dark pixels score 4. Both feet of all slaughtered broilers were measured and a mean value was calculated for each animal. Feet with a wrong position in the shackle or feathers on the footpads were excluded from the calculation. The total score of one flock was calculated: Flock footpad score = (0 x the total number of feet with score 1) + (0.5 × the total number of feet with score 2) + (2.0 x the total number of feet with score 3) + (2.0 × the total number of feet with score 4). The footpad score of the broilers in C1 was 13 and in C2 it was 6, resulting in a mean score of 9.5 for the whole flock. In comparison, the mean flock footpad score of 7 cycles before and 4 cycles after the EC infected cycle was 31.2 with every score of the different cycles lying clearly above 9.5 (Table 3).

**Table 3:** **Broiler footpad scores from different cycles of the farm with the EC outbreak, determined at the slaughterhouse** ^**A**^

**Broiler cycle**	**Score** ^**B**^ **compartment 1**	**Score** ^**B**^ **compartment 2**	**Mean score** ^**B**^ **compartments 1 + 2**
−7	28	16	22.1
−6	35	29	32.0
−5	16	11	13.5
−4	38	8	22.9
−3	50	81	65.5
−2	61	39	50.1
−1	31	35	33.0
0^C^	13	6	9.5
+1	34	16	25.8
+2	30	31	30.5
+3	24	22	23.0
+4	25	24	24.5
**Mean footpad score all cycles without cycle 0**			**31.2**

^A^Assessed with an automated camera system.

^B^The assessment is based on color and size of the lesions. The area of the foodpad was determined and divided into pixels. The proportion of dark skin (representing foot pad lesions) within the area designated as the footpad was measured and used for categorizing the severity of footpad dermatitis. Foot pads were scored according to the number of dark pixels from score 1 to score 4, with 0–5 dark pixels as score 1, 6–19 dark pixels score 2, 20–44 dark pixels score 3 and 45–100 dark pixels score 4. Both feet of all slaughtered broilers were measured and a mean value was calculated for each animal. The total score of one flock was calculated: Flock footpad score = (0 x the total number of feet with score 1) + (0.5 x the total number of feet with score 2) + (2.0 x the total number of feet with score 3) + (2.0 x the total number of feet with score 4).

^C^Broiler cycle with the EC outbreak. Cycles before and after the EC infection are labeled with “-“ or “+” respectively.

### Histopathology

**Figure 5:**
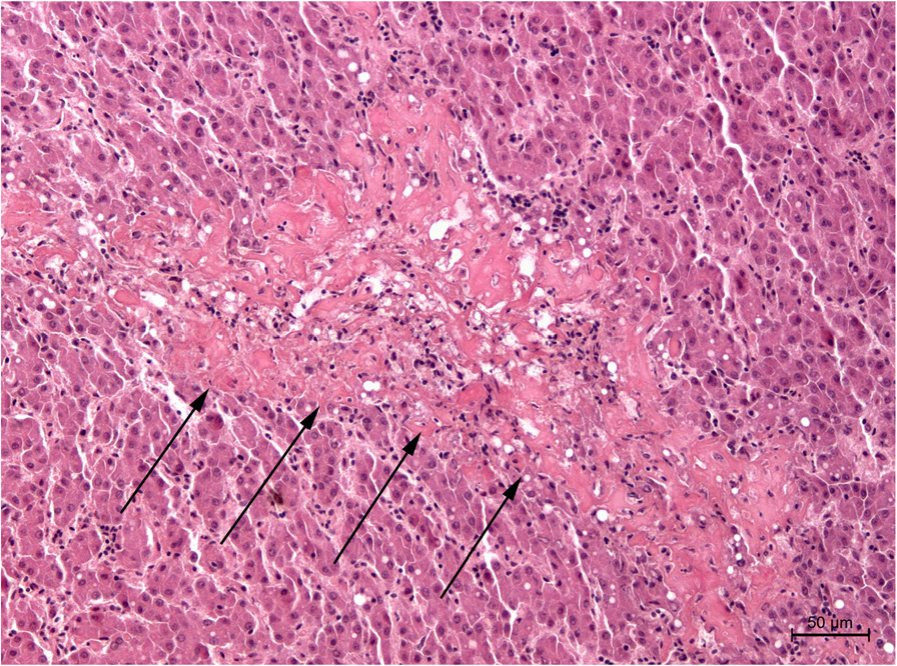
**Histopathologic changes in the liver of an** ***Enterococcus cecorum*** **infected broiler, hematoxylin and eosin staining.** A large area of necrosis (arrows) consisting of eosinophilic, proteinaceous material, 200-fold magnification.

Heart and liver of two EC-positive animals from day 17 ph were fixed in 10% phosphate-buffered formalin for at least 24 hours, dehydrated in graded alcohols and embedded in paraffin. Tissue sections of 2 μm were stained with hematoxylin and eosin. Histopathological examination of the heart and pericardium showed pericarditis and epicarditis. The thickness of the pericardium and epicardium was increased and in the pericardial cavity, masses of amorphous fibrinous material mixed with heterophilic and mononuclear cells were detected. Furthermore, multifocally distributed heterophils, macrophages and lymphocytes were found throughout the pericardium and epicardium. Hepatitis was found in the liver with multifocal necrotic foci composed of necrotic hepatocytes and fibrinous exudate (Figure 5).

### Bacteriology

Heart, liver, spleen and, where applicable, swab samples of the yolk sac and vertebral column were collected from the examined animals and inoculated onto Columbia Sheep Blood (CSB) agar and Cystine Lactose Electrolyte Deficient (CLED) agar plates (Oxoid GmbH, Wesel, Germany) and incubated for 24 hours at 37°C under microaerophilic conditions. After 24 hours, agar plates were monitored for bacterial growth, results were documented and pure subcultures from single colonies were produced on CSB agar. On the following day, subcultures were processed for Gram staining, catalase reaction and identification with the commercial micro-organism identification system rapid ID 32 STREP (BioMerieux, Nuertingen, Germany) were conducted following manufacturer’s instructions. Organs from 59 broilers were subjected to bacteriological analysis and EC was isolated from 23 birds (39.0%). In total, 59 hearts, 59 livers, 55 spleens, 4 yolk sacs and 4 swabs from the vertebral column were examined. EC was isolated as pure cultures from all EC positive animals apart from one broiler at 24 dph, which was also positive for *E. coli*. The first EC-positive broiler was found at 3 dph, EC was isolated from the yolk sac in high numbers (Table 1). At 6 dph, EC was already isolated from heart, liver and spleen of systemically infected broiler chickens. EC was detected in heart, liver and spleen of several broilers at every date of examination until the end of the cycle (Table 1) and was also isolated from the spinal column of one animal with vertebral osteomyelitis. *E. coli* was isolated from 11 broilers of both compartments (18.6%), predominantly from 3 to 13 dph (data not shown).

Antimicrobial susceptibility was tested for 4 isolates from days 3, 6 and 10 ph using the agar disc diffusion method. Bacterial isolates were cultured on Mueller-Hinton agar plates with added sheep blood (Oxoid GmbH) and inoculated for 24–28 h at 37°C before diameters of inhibition zones were evaluated and interpreted according to the Clinical and Laboratory Standards Institute guidelines [22]. Following antibiotic substances were tested (μg per disc; Oxoid GmbH; tylosin: MAST Diagnostica GmbH, Reinfeld, Germany): sulfamethoxazol/trimetoprim (25), enrofloxacin (5), neomycin (10), oxytetracycline (30), colistin sulfate (10), amoxycillin (10), sulfonamid (300), ampicillin (10), erythromycin (15), clindamycin (2), lincomycin (10), tylosin (30) and doxycycline (30). All 4 isolates were resistant against colistin and sulfonamid, 3 isolates were resistant against sulfamethoxazol/trimethoprim and neomycin. All isolates were sensitive to all other tested substances.

### Molecular identification

The results of the phenotypic identification methods were verified for 3 representative EC isolates using 16S-rRNA partial gene sequencing. Briefly, bacterial DNA of the subcultures was isolated using a commercially available mini spin filter system (innuPrep bacteria DNA kit; Analytic Jena, Jena, Germany). A 440-bp gene segment of the 16S rRNA was amplified using primers 91E_for (GGAATTCAAAKGAATTGACGGGGGC), 13B_rev (CGGGATCCCAGGCCCGGGAACGTATTCAC) and conditions according to Mignard and Flandrois [23]. PCR products were sequenced at Seqlab (Göttingen, Germany). DNA sequence analysis was performed using the BLAST database of the American National Center for Biotechnology Information (Bethesda, Maryland, USA) and the EzTaxon-e server [24], which contains only type strains. The phenotypic identification results of 3 EC isolates could be verified with 16S-rRNA partial gene sequencing. Sequences are available in Genbank under accession numbers KJ909206-KJ909208.

## Discussion

This case report describes for the first time an investigation of an EC-infected broiler flock over the course of the whole growing period. All other available reports selected and examined broilers from one specific date during the rearing period [10,12,13,15,16] and therefore may have missed important factors concerning the pathogenesis of EC infection in these animals. In most reports, broilers were not submitted for necropsy before the flock showed lameness, maybe missing EC isolations from internal organs like heart and liver as potential initial stages of EC infection and giving no information about the progression of the infection.

The natural infection route of EC in broilers is not known. A generalized EC infection was recently reported in a pigeon with lesions in the ventriculus, suggesting an invasion of EC via the digestive tract [25]. Another possible route via the respiratory tract has also been suggested for EC in broilers [26]. Furthermore, EC associated disease was reproduced in Pekin ducks by inoculation via air sacs [20]. In Germany, EC outbreaks in broilers were frequently observed in flocks with high amounts of dust in the air, especially in flocks with underfloor heating systems (personal communication with other poultry veterinarians). An infection via the respiratory system with EC attached to dust particles seems very likely in this context. Several possible reasons for the sudden appearance of EC as a disease causing agent in broilers were discussed [12], but never the litter management. At least in Germany, a great deal of effort was expended in the last recent years to maintain dry litter throughout the whole broiler cycle, basically because wet litter is the main cause for footpad dermatitis. Footpad monitoring programs at broiler slaughterhouses with monetary consequences for the farmers probably lead to dryer litter throughout the cycles. But dry litter means also more dust. The theory of EC infection as an airborne disease is supported by data from the investigated farm. The investigated cycle with the EC outbreak had by far the best footpad score at the slaughterhouse compared to 7 cycles before and 4 cycles after the EC infection. However, air quality in the broiler house was not monitored and EC concentration in the dust was not measured. The broiler cycle with the EC outbreak was treated similar to the other cycles, no particular preventive measures to avoid footpad lesions were conducted by the farmer. Although there are some indications for the theory of airborne infection of EC, we cannot exclude the possibility that the low footpad scores are somehow a consequence of the EC infection or that these scores were just by chance the best ones in the monitored growing cycles.

EC is the dominating *Enterococcus* species in layers and broiler parents older than 10–12 weeks, but has also been found in the intestine of younger birds [9,27]. If chickens are colonized with EC, it can be assumed that EC is also shed in high amounts in the faeces and is therefore also attached to dust particles in the broiler house. In this study, EC was already isolated from the yolk sac of a broiler at 3 dph. There were no clinical signs of disease in the flock at that time. To date, vertical transmission of EC has not been clearly demonstrated, nor has EC been found in air samples of the hatchery [13,15]. Potential reservoirs at the farm including the water system or rodents also had negative test results for EC [15]. Nevertheless, EC-associated disease was reported from multiple successive rounds of the same broiler house, indicating an on farm reservoir as a source of EC infection [12]. Apart from this report, EC was isolated from a yolk sac remnant of a broiler in one other study [10] and from 7 of 10 examined yolk sacs of Pekin ducklings with early mortality [18]. Thus, navel and yolk sac infection may play also a role in the pathogenesis of EC infections in broilers. Remarkably, pericarditis and hepatitis were frequently found in EC-infected broilers in our study, preceding osteomyelitic changes which emerged later in the course of infection. Because of our observations, we assume that bacteraemia and generalization are crucial steps in the pathogenesis of EC infection in broilers. Pericarditis and hepatitis were found as early as 6 dph, long before femoral head necrosis and vertebral osteomyelitis appeared, and EC was isolated continuously from these organs. Pericarditis was also described in some of the other case reports dealing with EC infection in broilers [10,13,14], hepatitis was reported sporadically in one study [13] and indirectly in a second publication, where EC was detected as coccoid bacteria in the histological examination of the liver [12]. Pericarditis and hepatitis may have been missed in other case reports, because they are not as obvious as for example pathological changes during *E. coli* infection. Our results emphasize the importance of high quality veterinary support and thoroughly performed pathological and bacteriological examinations for the early diagnosis of EC infections in broiler flocks. Early diagnosis may be crucial for successful antibiotic therapy, which has to be administered during the initial stages of the disease [10]. In this report as well as in other studies, antibiotic treatment of broilers with an advanced stage of EC infection had only temporary effects or no success at all [10,13], although no resistance against the used antibiotic substance was detected in 4 tested EC isolates in this report.

The overall mortality rate in the described flock was 7.22% within 33 days. The mortality rate of the *E.-coli*-vaccinated compartment 1 was 5.84% and 8.59% in compartment 2. Additionally, *E. coli* was isolated from some broilers up to day 13 ph. Therefore, a part of the early mortality, which was already lower in compartment C1 during the first week post hatch, was likely due to an *E. coli* infection as it mainly occurred before the development of vaccinal immunity against this bacterium.

Higher mortality rates up to 15% were reported from EC infected broiler flocks with longer rearing periods [15,21], but flock mortality represents only one part of the economic impact of EC infection. In the current case, 9.75% (C1: 3.33%, C2: 6.42%) of the slaughtered broilers were rejected at the slaughterhouse, which is even higher than the mortality rate. The mean percentage of rejected broilers in the last 5 cycles of the farm was 2.23% in comparison. Most of the chickens were rejected due to cellulitis (5.96%) and generalized disease (2.75%). *Escherichia coli* has been reported to be the predominant microorganism isolated from cellulitis lesions of broiler chickens [28-30]. It can be speculated that scratching of lame animals favoring the development of cellulitis. Therefore, EC may be considered as the underlying cause for the rejected animals.

This report describes an example of EC associated disease in broilers that resulted in economic losses for the farmer due to increased mortality, the costs of therapy and increased condemnations at the slaughterhouse. Condemnations due to cellulitis induced by the scratching of lame birds may have further increased the economic impact of this condition.

## Conclusions

This report describes for the first time the whole course of disease of an *Enterococcus cecorum* infected broiler flock over the entire growing cycle. Bacteraemia and generalization seem to be important steps in the pathogenesis of *Enterococcus cecorum* infection in broilers, preceding frequently reported femoral head necrosis and vertebral osteomyelitis. The route of infection may be via the respiratory tract with EC attached to dust particles, but further research is needed to confirm this theory. *Enterococcus cecorum* associated disease in broilers impacts the farmers economically, not only as a result of elevated flock mortality but also because of higher condemnation rates at the slaughterhouse.

### Availability of supporting data

The 16S-rRNA partial gene sequences from 3 EC isolates are available via the following links:

• http://www.ncbi.nlm.nih.gov/nuccore/KJ909206

• http://www.ncbi.nlm.nih.gov/nuccore/KJ909207

• http://www.ncbi.nlm.nih.gov/nuccore/KJ909208
